# Eicosanoid Derivative, Lipoxin A4, Guards Against Testicular Ferroptosis in Rat Model of Type II Diabetes by Regulating Nrf2/SLC7A11/GPX4 Pathway

**DOI:** 10.3390/ijms27083548

**Published:** 2026-04-16

**Authors:** Elshymaa A. Abdel-Hakeem, Manar Fouli Gaber Ibrahim, Doaa Mohamed Elroby Ali, Shimaa Abdel Baset Abdel Hakim, Ahmed M. Ashour, Ali Khames, Heba A. Abdel-Hamid

**Affiliations:** 1 Department of Medical Physiology, Faculty of Medicine, Minia University, Minia 61111, Egypt; shady@mu.edu.eg (E.A.A.-H.); heba.abdelhameed@minia.edu.eg; 2 Department of Histology and Cell Biology, Faculty of Medicine, Minia University, Minia 61111, Egypt; manar.fouli@mu.edu.eg; 3 Faculty of Physical Therapy, Lotus University, Minia 61768, Egypt; 4 Department of Histology and Cell Biology, Faculty of Medicine, Minia National University, Minia 61111, Egypt; 5 Department of Biochemistry, Faculty of Pharmacy, Sohag University, Sohag 82524, Egypt; doaa.elroby@pharm.sohag.edu.eg; 6 Department of Biochemistry, Faculty of Pharmacy, Badr University Branch Assiut, Assiut 71511, Egypt; 7 Department of Anatomy, Faculty of Medicine, Minia University, Minia 61111, Egypt; shimaa.abdelbaset@mu.edu.eg; 8 Department of Pharmacology and Toxicology, College of Pharmacy, Umm Al-Qura University, Makkah 21955, Saudi Arabia; 9 Department of Pharmacology and Toxicology, Faculty of Pharmacy, Sohag University, Sohag 82524, Egypt; 10 Department of Medical Physiology, Faculty of Medicine, Al-Baha University, Al-Baha 65779, Saudi Arabia

**Keywords:** lipoxin A4, diabetes, rats, testis, ferroptosis, iron

## Abstract

Ferroptosis, a type of iron overload-induced cell death, is involved in diabetes-induced testicular dysfunction. Hence, this study was designed to investigate, for the first time, the impact of lipoxin A4 (LXA4) administration on testicular tissue in diabetic rats and explore its probable role in regulating ferroptosis in comparison with the standard ferroptosis inhibitor (ferrostatin-1, Fer-1). Albino rats of Wistar strain were divided into a control group, a type II diabetes mellitus (DM) group, a DM + Fer-1group, and a DM + LXA4 group. Serum levels of iron, insulin, glucose, total cholesterol, triglycerides, and testosterone were assayed. Testicular tissue markers of oxidative stress, ferroptosis, and inflammation were also assessed by different methods. Our results confirmed diabetes-induced testicular injury and disruption of its function via inducement of ferroptosis, but this was ameliorated with LXA4 and Fer-1 administration. However, Fer-1 showed a greater protective effect compared to LXA4 under the conditions of this study. We concluded that LXA4 partially secured the testicular tissue of diabetic rats against ferroptosis via augmenting the antioxidant Nrf2/SLC7A11/GPX4 pathway. Therefore, LXA4 may have a possible protective effect on the testicular tissue of diabetic patients.

## 1. Introduction

Diabetes mellitus can cause serious complications to all organs of the body, including the testes, which may affect male fertility. However, the mechanisms of these complications are still unclear [1]. A previous study documented that the development of diabetic complications is closely related to a new type of cell death not due to apoptosis, but due to iron accumulation within the tissue, which is called ferroptosis [2]. A recent study showed that ferroptosis participated in the pathogenesis of diabetic testicular disorders [3]. Iron accumulation in the testes could lead to oxidative stress, resulting in ferroptosis and hence testicular dysfunction. But this effect can be corrected using iron chelators or ferroptosis inhibitors such as ferrostatin-1 (Fer-1) [4]. This indicates that ferroptosis may be considered a therapeutic target for the treatment of testicular disorders on top of diabetes [5].

Nuclear factor erythroid 2-related factor 2 (Nrf2) is a transcription factor that plays a critical role in mitigating intracellular oxidative stress through the expression of several antioxidants, including solute carrier family 7 member 11 (SLC7A11) and glutathione peroxidase 4 (GPX4), the main constituents of the ferroptosis pathway [6]. SLC7A11 is an amino acid anti-transporter protein that plays a vital role in inhibiting ferroptosis by facilitating cystine uptake for glutathione (GSH) synthesis, thereby enhancing the cell’s antioxidant capacity. GPX4 prevents ferroptosis by reducing lipid hydroperoxides to lipid alcohols, leading to inhibition of ROS accumulation [7]. Several studies reported that the Nrf2/SLC7A11/GPX4 pathway attenuated lipid peroxidation and ferroptosis in testicular tissues of diabetic rats [5,7,8].

Lipoxin A4 (LXA4), a naturally occurring eicosanoid, is an omega-6 polyunsaturated fatty acid with potent antioxidant and anti-inflammatory actions and is generated endogenously from arachidonic acid. A previous study reported that the endogenous LXA4 plasma level was reduced in diabetics, and its exogenous administration improved insulin resistance and prevented the occurrence of diabetes mellitus (type 2) both in animals and humans [9]. In addition, it can prevent several diabetic complications, including vascular complications, nephropathy [10], retinopathy [11], and erectile dysfunction [12]. But no study till now has addressed the influence of LXA4 on diabetic testis and its role in the regulation of ferroptosis. Thus, this research was planned to study the effect of exogenous LXA4 administration on testicular tissues of diabetic rats and its role in the regulation of ferroptosis in comparison with the ferroptosis inhibitor, Fer-1, focusing on the Nrf2/SLC7A11/GPX4 pathway.

## 2. Results

### 2.1. Evaluation of Glycemia, Total Cholesterol and Triglycerides

As seen in Table 1, the serum levels of glucose, total cholesterol, and triglycerides were significantly elevated, while the serum insulin level was significantly dropped in the diabetic group when compared with the control group. Administration of Fer-1 and LXA-4 significantly decreased these elevated parameters and improved the insulin level, but they were still significantly different from the normal control levels. However, Fer-1 produced significantly greater improvement than LXA4.

### 2.2. Evaluation of Serum Iron and Testicular Tissue Levels of Iron and Ferritin

Both serum and testicular iron were significantly increased, while the testicular ferritin level was significantly decreased in the diabetic group in comparison with the control group. Following administration of Fer-1 and LXA4, these parameters were improved, but they were still significantly different from the control levels. Nevertheless, Fer-1 produced significantly greater improvement than LXA4 (Table 2).

### 2.3. Evaluation of Sperm Count, Motility and Serum Testosterone Level

**Figure 1:**
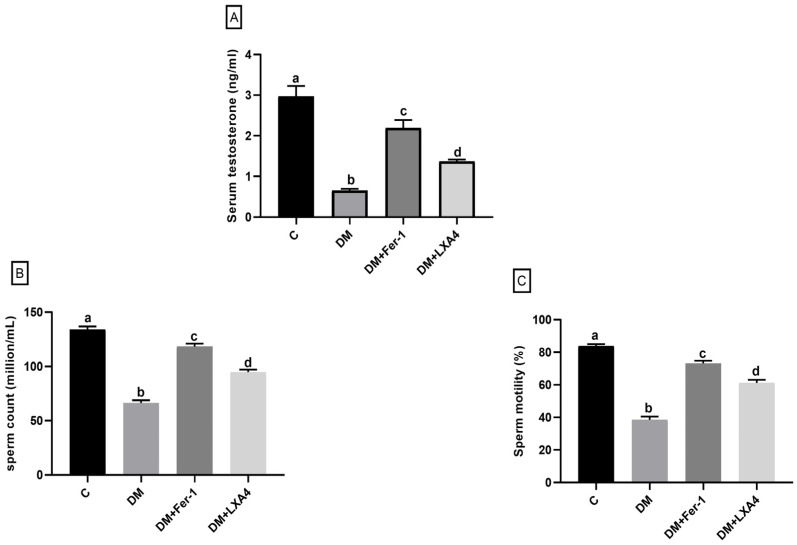
Changes in serum testosterone level (**A**), sperm count (**B**) and sperm motility (**C**) of different groups. Values of six rats in each group are expressed as mean ± SEM. Different letters (a, b, c and d) above the bars indicate significant differences between groups (*p* value ≤ 0.05). Pairwise comparisons using Tukey’s post hoc test revealed significant differences between all groups. All comparisons (*p* < 0.0001) except for testosterone; a versus c (*p* = 0.02), b versus d (*p* = 0.006) and c versus d (*p* = 0.005), sperm count; a versus c (*p* = 0.001), and sperm motility; a versus c (*p* = 0.02), and c versus d (*p* = 0.003). C: control; DM: type II diabetes group; Fer-1: ferrostatin-1; LXA4: lipoxin A4.

Regarding testosterone level, sperm count and motility, they were significantly dropped in the diabetic group in comparison with the control one. Upon administration of Fer-1 and LXA4, these parameters were significantly enhanced, but they were still significantly different from the control level. Notably, Fer-1 produced significantly greater improvement than LXA4 (Figure 1).

### 2.4. Evaluation of Testicular Oxidant and Inflammatory States

The oxidative stress markers (ROS and MDA) and the inflammatory markers (NF-κB p65 and TNF-α) were significantly elevated, while the antioxidants (GPX4 and GSH) and the anti-inflammatory cytokines (IL-37) were significantly decreased in the diabetic group when compared with the control one. Notably, these parameters were reversed upon administration of Fer-1 and LXA4, but they were still significantly different from the control level. Fer-1 was associated with significantly greater improvement than LXA4 (Table 3).

### 2.5. Results of RT-PCR Assessment of GPX4

**Figure 2:**
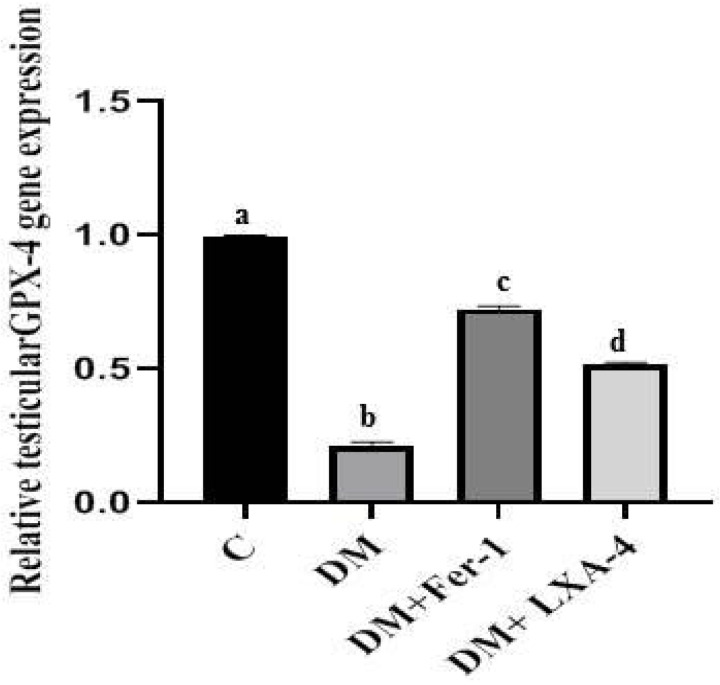
Gene expression of GPX4 of different groups. Values of six rats in each group are expressed as mean ± SEM. Different letters (a, b, c and d) above the bars indicate significant differences between groups (*p* ≤ 0.05). All pairwise comparisons using Tukey’s post hoc test revealed significant differences between all groups (*p* < 0.0001). C: control; DM: type II diabetes group; Fer-1: ferrostatin-1; LXA4: lipoxin A4; GPX4: glutathione peroxidase 4.

The mRNA level of GPX4 was significantly reduced in the diabetic group in comparison with the control one (*p* < 0.0001). Administration of Fer-1 and LXA4 led to an elevation of the mRNA level of GPX4, but it was still significantly different from the control level. Fer-1 significantly increased the mRNA level of GPX4 more than LXA4 (Figure 2).

### 2.6. Results of Western Blotting Analysis of Nrf2 and SLC7A11

**Figure 3:**
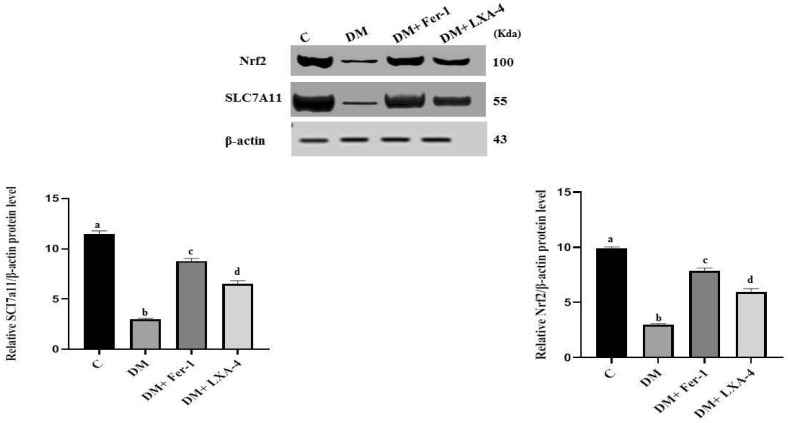
Western blotting analysis of Nrf2 and SLC7A11 protein levels in different groups. Values of six rats in each group are expressed as mean ± SEM. Different letters (a, b, c and d) above the bars indicate significant differences between groups (*p* ≤ 0.05). All pairwise comparisons using Tukey’s post hoc test revealed significant differences between all groups (*p* < 0.0001). C: control; DM: type II diabetes group; Fer-1: ferrostatin-1; LXA4: lipoxin A4; Nrf2: nuclear factor erythroid 2-related factor 2; SLC7A11: solute carrier family 7 member 11.

The protein levels of both Nrf2 and SLC7A11 were significantly dropped in the diabetic group in comparison with the control group. Administration of Fer-1 and LXA4 reversed this effect and led to elevation of these protein levels, but they were still significantly different from the control level. However, Fer-1 significantly increased these protein levels more than LXA4 (Figure 3).

### 2.7. Histological Results

#### 2.7.1. Hematoxylin and Eosin (H&E) Results

**Figure 4:**
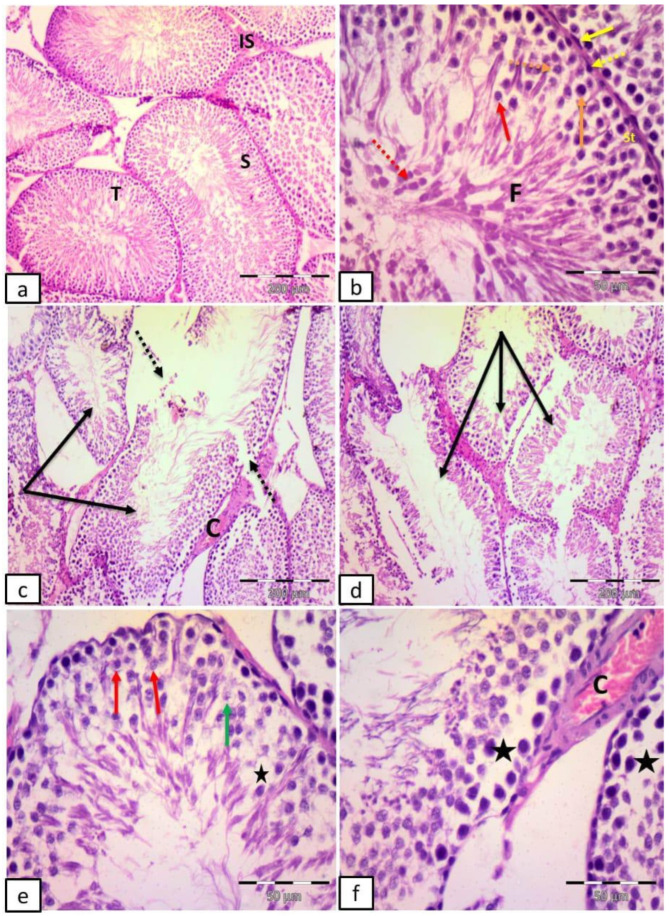
Representative photomicrographs of testicular sections in adult male albino rats. (**a**,**b**) Control group: multiple seminiferous tubules (T) with regular outlines, lined by stratified germinal epithelium (S) and narrow interstitial spaces (IS). Notice Sertoli cells (St), spermatogonia (dotted yellow arrow) resting on the basement membrane, 1ry spermatocyte with large nuclei containing dark clumps of chromatin (orange arrow), 2ry spermatocytes (dotted orange arrow), rounded spermatids with rounded nuclei (red arrow), late spermatids (dotted red arrow) with elongated nuclei, flagella of mature sperms in the lumen of the tubules (F), and flat myoid cells with flattened nucleus (yellow arrow) surrounding the tubules. (**c**–**f**) Group II (DM): many distorted tubules (black arrows), discontinuous basement membrane (dotted arrows), empty spaces in between germ cells (stars), vacuolated cells (red arrows) and ghosts of nuclei (green arrow). Notice congested blood vessels (C) in the interstitial space. (H&E (**a**,**c**,**d**) ×100; scale bar = 200; (**b**,**e**,**f**) ×400; scale bar = 50 µm).

The control group revealed multiple seminiferous tubules with regular outlines, lined by stratified germinal epithelium and narrow interstitial spaces. Sertoli cells, spermatogonia resting on the basement membrane, 1ry spermatocyte with large nuclei containing dark clumps of chromatin, 2ry spermatocytes, rounded spermatids with rounded nuclei, late spermatids with elongated nuclei, flagella of mature sperms in the lumen of the tubules, and flat myoid cells with flattened nucleus surrounding the tubules were noticed (Figure 4a,b).

Group II (DM) showed many distorted tubules, discontinuous basement membrane, empty spaces in between germ cells, vacuolated cells and ghosts of nuclei. Congested blood vessels in the interstitial space were also noticed (Figure 4c–f).

**Figure 5:**
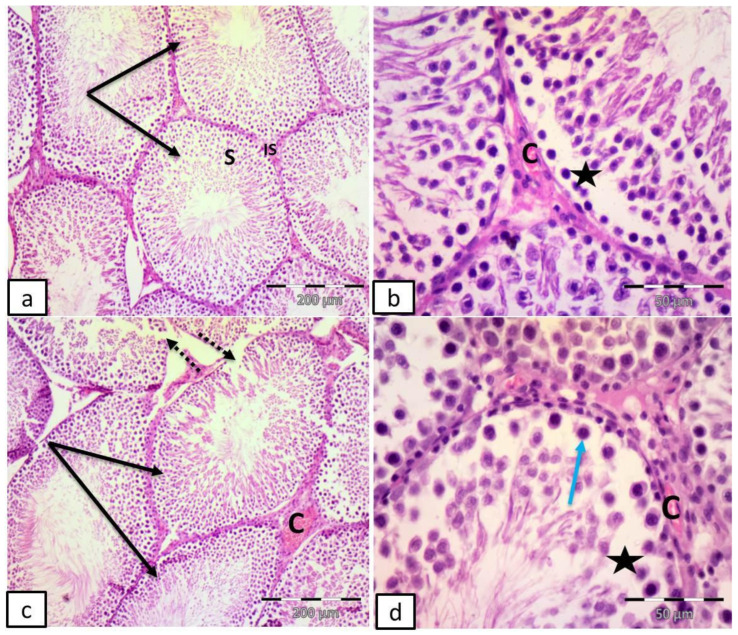
Representative photomicrographs of testicular sections in adult male albino rats. (**a**,**b**) Group III (DM + Fer-1): apparent restoration of the seminiferous tubules with regular outlines (black arrows), lined by stratified germinal epithelium (S) with narrow interstitial spaces (IS). Notice empty spaces in-between germinal epithelium (stars), and congested blood vessels (C). (**c**,**d**) Group IV (DM + LXA4); multiple seminiferous tubules with apparently regular outlines (black arrows). Notice discontinuous basement membrane (dotted arrows), empty spaces in between germ cells (stars), vacuolated cells (blue arrow) and congested blood vessels (C). (H&E (**a**,**c**) ×100; scale bar = 200; (**b**,**d**) ×400; scale bar = 50 µm).

Group III (DM + Fer-1) showed apparent restoration of the seminiferous tubules with regular outlines, lined by stratified germinal epithelium with narrow interstitial spaces. Empty spaces in between the germinal epithelium and congested blood vessels were also revealed (Figure 5a,b).

Group IV (DM + LXA4) revealed multiple seminiferous tubules with apparently regular outlines, discontinuous basement membrane, empty spaces in between germ cells, vacuolated cells and congested blood vessels (Figure 5c,d).

#### 2.7.2. Perl’s Prussian Blue Stain Results

**Figure 6:**
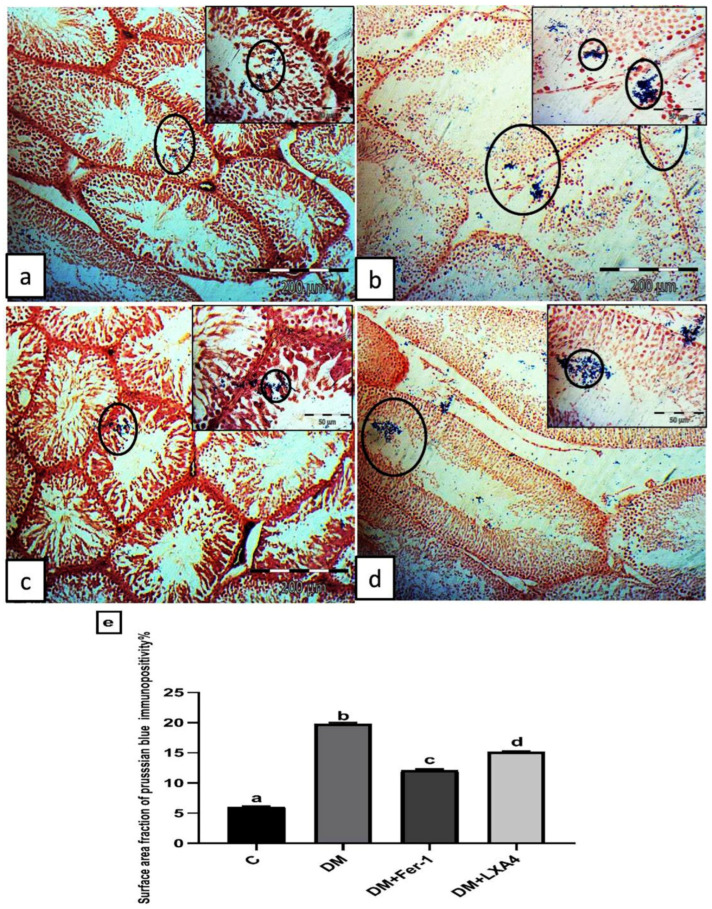
Representative photomicrographs of testicular sections stained by Perl’s Prussian blue stain. (**a**) Control group: minimal Perl’s Prussian blue-stained depositions within the seminiferous tubules (circles). (**b**) Group II (DM): excessive deposition within the seminiferous tubules and interstitial spaces (circles). (**c**) Group III (DM + Fer-1): little deposition (circles) within the seminiferous tubules. (**d**) Group IV (DM + LXA4): some deposition (circles) within the seminiferous tubules (Perl’s Prussian blue × 100; scale bar = 200 µm; insert × 400; scale bar = 50 µm). (**e**) Histogram showing the surface area fraction of Prussian blue immunopositivity in all studied groups. Values of six rats in each group are expressed as mean ± SEM. Different letters (a, b, c and d) above the bars indicate significant differences between groups (*p* ≤ 0.05). All pairwise comparisons using Tukey’s post hoc test revealed significant differences between all groups (*p* < 0.0001). C: control; DM: type II diabetes group; Fer-1: ferrostatin-1; LXA4: lipoxin A4.

The control group revealed minimal Perl’s Prussian blue-stained depositions within the seminiferous tubules (Figure 6a). The DM group revealed excessive deposition within the seminiferous tubules and interstitial spaces (Figure 6b). Group III (DM + Fer-1) revealed little deposition within the seminiferous tubules (Figure 6c). Group IV (DM + LXA4) revealed some depositions within the seminiferous tubules (Figure 6d). The surface area fraction of Prussian blue immunopositivity was significantly elevated in the DM group in comparison with the control one. This effect was reversed in the DM + Fer-1 and DM + LXA4 groups, but much more in the DM + Fer-1 group (Figure 6e).

#### 2.7.3. Immunohistochemistry Results for Anti-HMGB1 Antibody

**Figure 7:**
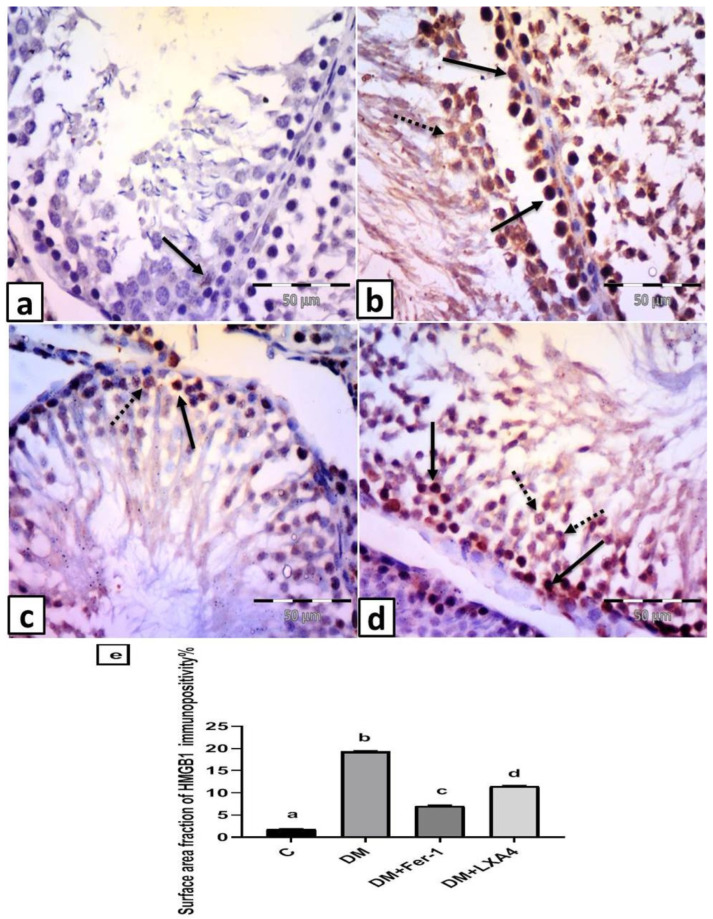
Representative photomicrographs of testicular sections immunolabelled by HMGB1. (**a**) Control group: scattered cells with faint nuclear expression in testicular cells (arrow). (**b**) Group II (DM): numerous cells with positive cytoplasmic (dotted arrow) or nuclear and cytoplasmic (arrows) expression. (**c**) Group III (DM + Fer-1): some cells with positive cytoplasmic (dotted arrow) or nuclear and cytoplasmic (arrow) expression. (**d**) Group IV (DM + LXA4): many cells with positive cytoplasmic (dotted arrows) or nuclear and cytoplasmic (arrows) expression. (HMGB1 × 400; scale bar = 50 µm). (**e**) Histogram showing the surface area fraction of HMGB1 immunopositivity in all studied groups. Values of six rats in each group are expressed as mean ± SEM. Different letters (a, b, c and d) above the bars indicate significant differences between groups (*p* value ≤ 0.05). All pairwise comparisons using Tukey’s post hoc test revealed significant differences between all groups (*p* < 0.0001). C: control; DM: type II diabetes group; Fer-1: ferrostatin-1; LXA4: lipoxin A4.

The control group revealed scattered cells with faint nuclear expression in testicular cells (Figure 7a). Group II (DM) revealed numerous cells with positive cytoplasmic or nuclear and cytoplasmic expression (Figure 7b). Group III (DM + Fer-1) showed some cells with positive cytoplasmic or nuclear and cytoplasmic expression (Figure 7c). Group IV (DM + LXA4) revealed many cells with positive cytoplasmic or nuclear and cytoplasmic expression (Figure 7d). The surface area fraction of HMGB1 immunopositivity was significantly elevated in the DM group in comparison with the control one. This effect was reversed in the DM + Fer-1 and DM + LXA4 groups, but much more in the DM + Fer-1 group (Figure 7e).

#### 2.7.4. Immunohistochemistry Results for Anti-Tunnel Antibody

**Figure 8:**
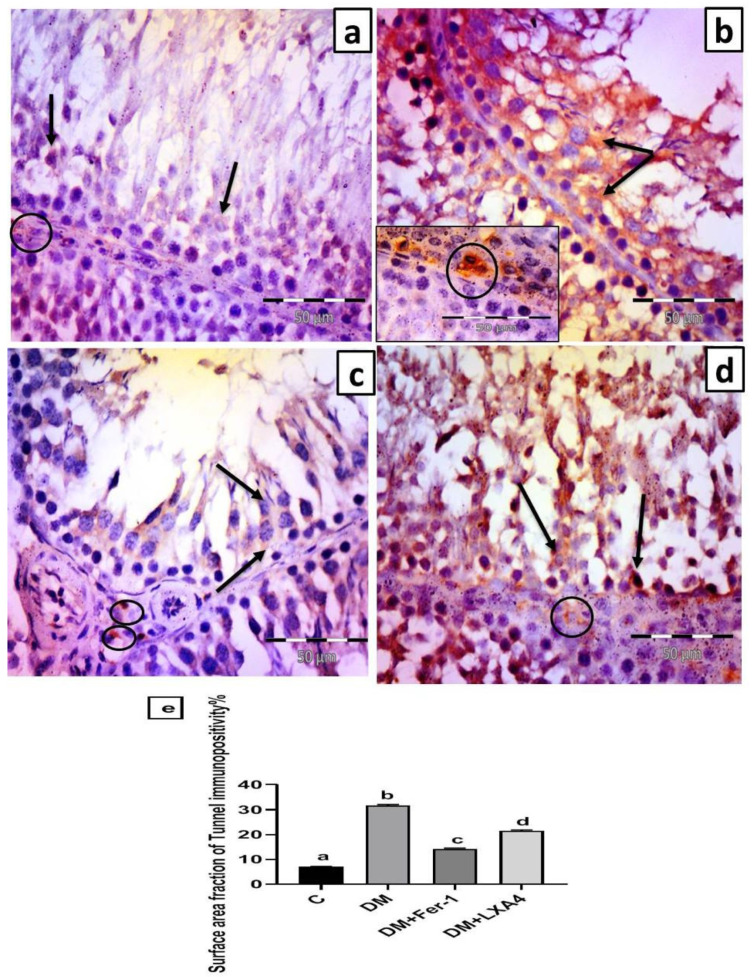
Representative photomicrographs of testicular sections immunolabelled by Tunnel. (**a**) Control group: scattered cells with faint positive cytoplasmic expression in germinal epithelium (arrow) and faint positive cytoplasmic expression in Leydig cells (circle). (**b**) Group II (DM): strong positive cytoplasmic expression in germinal epithelium (arrow) and Leydig cells (circle). (**c**) Group III (DM + Fer-1): some germinal epithelial cells with faint positive cytoplasmic expression (arrows). Notice scattered Leydig cells with strong cytoplasmic expression (circles). (**d**) Group IV (DM + LXA4): many germinal epithelial cells with strong positive cytoplasmic expression (arrows) and some Leydig cells with strong cytoplasmic expression (circle). (Tunnel × 400; scale bar = 50 µm). (**e**) Histogram showing the surface area fraction of Tunnel immunopositivity in all studied groups. Values of six rats in each group are expressed as mean ± SEM. Different letters (a, b, c and d) above the bars indicate significant differences between groups (*p* value ≤ 0.05). All pairwise comparisons using Tukey’s post hoc test revealed significant difference between all groups (*p* < 0.0001). C: control; DM: type II diabetes group; Fer-1: ferrostatin-1; LXA4: lipoxin A4.

The control group revealed scattered cells with faint positive cytoplasmic expression in the germinal epithelium and faint positive cytoplasmic expression in Leydig cells (Figure 8a). Group II (DM) revealed strong positive cytoplasmic expression in germinal epithelial cells and Leydig cells (Figure 8b). Group III (DM + Fer-1) revealed some germinal epithelial cells with faint positive cytoplasmic expression and scattered Leydig cells with strong cytoplasmic expression (Figure 8c). Group IV (DM + LXA4) revealed many germinal epithelial cells with strong positive cytoplasmic expression and some Leydig cells with strong cytoplasmic expression (Figure 8d). The surface area fraction of Tunnel immunopositivity was significantly elevated in the DM group in comparison with the control one. This effect was reversed in the DM + Fer-1 and DM + LXA4 groups, but much more in the DM + Fer-1 group (Figure 8e).

## 3. Discussion

Testicular ferroptosis on top of diabetes is characterized by a higher amount of free iron in testicular tissue, which generates reactive oxygen species (ROS), resulting in lipid peroxidation, depletion of antioxidants, and hence disruption of cell structure. Thus, using antioxidants in diabetic cases may be vital for the amelioration of testicular ferroptosis and the resulting male infertility [13].

In our study, we investigated the probable protective action of lipoxin A4 (LXA4) administration, a potent antioxidant and anti-inflammatory treatment, on testicular tissue of diabetic rats (type II diabetes) in comparison with ferrostatin-1 (Fer-1, a ferroptosis inhibitor). We found that the induction of type II diabetes led to testicular ferroptosis that disrupted the testicular structure and functions, both endocrine and spermatogenic [14]. This is in agreement with the elevated markers of ferroptosis, such as ROS, MDA, and iron, along with a positive Prussian blue stain that indicated excessive iron deposition within the seminiferous tubules and interstitial spaces of testicular tissue in the diabetic group of this study.

When diabetic rats were treated with LXA4 and Fer-1, these effects were ameliorated. But we found that the protective effect of Fer-1, the direct iron chelator, is more obvious than that of LXA4. This agrees with a certain study which found that ferroptosis negatively affected the testicular spermatogenic function in type I diabetic patients and mice, but this effect was reversed with Fer-1 administration [4]. Another study found that type 2 diabetes in mice resulted in testicular injury via augmenting the testicular tissue levels of ROS, lipid peroxides, and ferrous iron, but these effects were corrected with Fer-1 administration in combination with an oral hypoglycemic drug (Semaglutide) [14]. On the other hand, LXA4 administration may reduce testicular ferroptosis indirectly through its antioxidant effect, as evidenced in this study by the reduction in ROS, lipid peroxides, iron content, and elevation of ferritin content and antioxidants in testicular tissues. The antioxidant action of LXA4 is largely mediated through inhibiting the inflammatory processes that generate ROS and by activating endogenous antioxidant pathways [15]. This is compatible with the study of Li et al., who reported that LXA4 administration alleviated liver ferroptosis through its anti-inflammatory effect in a mouse model of sepsis-induced liver injury [16].

Ferritin is a crucial protein that stores iron inside cells and releases it in a controlled manner, acting as the body’s main intracellular iron reservoir. A high tissue iron level is often accompanied by the breakdown of ferritin (Ferritinophagy), which releases iron and catalyzes ROS generation and subsequently induces ferroptosis. Thus, a high ferritin level indicates high storage of iron and a low free ferrous iron level, which reduces ferroptosis as found with LXA4 administration [13], while ferritin degradation results in free iron accumulation in testicular tissue, which enhances ferroptosis as found in the non-treated diabetic group [17]. Additionally, the restoration of iron metabolism, as evident in the present study by the reduction in serum iron levels, observed with LXA4 administration, improved insulin secretion and blood glucose levels [2]. This is in keeping with a study which reported that systemic iron overload disrupts β-cell function and enhances the development of type II diabetes mellitus [18]. The restoration of the metabolic disturbances may provide additional support for the protective action of LXA4 on testicular tissue. Thus, improving metabolic health through dietary and lifestyle changes may help in the reduction in testicular ferroptosis [19].

The mechanism of the anti-ferroptosis action of LXA4 in the diabetic testis may involve the augmentation of the antioxidant Nrf2/SLC7A11/*GPX4* pathway. SLC7A11 and *GPX4* are the downstream targets of Nrf2 that were reduced in the Nrf2 knockout mice, as confirmed by a previous study [20]. SLC7A11 stimulates cellular cystine uptake that is necessary for glutathione (GSH) production to maintain cellular antioxidant capacity [21]. *GPX4* is essential in the prevention of ferroptosis and in the maintenance of cell survival using GSH for the reduction in intracellular peroxides. Previous studies reported that *GPX4* absence or dysfunction results in oxidative stress-induced ferroptosis [22]. Also, several studies documented the vital role of this antioxidant pathway in the availability of anti-inflammatory cytokines and antioxidants that maintained the redox status and provided resistance against testicular ferroptosis [5,7,8]. Moreover, several studies documented that LXA4, via *Nrf2* pathway augmentation, inhibited ferroptosis in different models of injury, such as lung ischemia–reperfusion injury in mice [23], Erastin (ferroptosis inducer)-induced ferroptosis of spinal cord cells [24], and knee osteoarthritis [25]. Moreover, the study of Pecchillo Cimmino and his colleagues reported that *SLC7A11* was upregulated with the stimulation of LXA4 receptors [26], while the study of Li et al. reported that LXA4 administration reduced lung ferroptosis in a mouse model of cigarette smoking-induced COPD via activation of *GPX4* and glutathione availability [27].

The inhibitory effects of LXA4 on testicular ferroptosis reduced the associated release of damaging products from the testicular cells suffering from ferroptosis, such as high-mobility group box chromosomal protein 1 (HMGB1). This protein is already found in the nuclei of testicular cells. But in association with ferroptosis, HMGB1 protein is translocated to the cytoplasm and enhances tissue inflammation and proinflammatory cytokine production via activation of the proinflammatory pathway, *NF-κB* [7,28]. This is compatible with the elevated levels of *NF-κB p56*, *TNF-α,* and immunoexpression of HMGB1 in the testicular tissue of the non-treated diabetic group, while these parameters were reversed with LXA4. This agrees with previous studies which reported that LXA4 reduced testicular inflammation in different models of injury through reduction in HMGB1 and *NF-κB p56* [29,30].

The upregulated Nrf2/SLC7A11/*GPX4* pathway with LXA4 in this study can protect and preserve testicular tissue and hence its function [7]. This agrees with the elevated serum testosterone level and the improved semen parameters regarding the sperm count and motility, along with the low surface area fraction of Tunnel immunopositivity seen in the rat group treated with LXA4, which indicated diminished lipid peroxidation and a decreased rate of cell death on top of ferroptosis, and hence the preservation of cells within the testis. This was confirmed by a previous study which reported that the *Nrf2* gene knockout mouse model had a low fertility rate and low sperm count and motility as a result of ferroptosis via inhibiting the SLC7A11/GSH/*GPX4* pathway [31]. Moreover, a certain study reported that the reduction in GPX4 levels in testicular tissue resulted in testicular ferroptosis, sperm abnormalities, and oligospermia, ultimately leading to infertility, while elevating GSH levels mitigated these effects [32]. Therefore, targeting the Nrf2/SLC7A11/*GPX4* pathway may be considered a therapeutic target for the correction of ferroptosis and the maintenance of male fertility [33].

All previous effects illuminate that Fer-1 and LXA4 administration improved testicular structure and functions via their inhibitory effect on ferroptosis. But herein, the protective effect of Fer-1 was more pronounced than that of LXA4 under the conditions of this study. This notion from our point of view may be adjusted using a higher dose of LXA4 or different administration regimens in future studies, taking into consideration the results of previous studies in different models of injury which reported that the protective effect of LXA4 is dose-dependent [34,35].

## 4. Materials and Methods

### 4.1. Animals and Ethics

Twenty-four adult (6–8 weeks) albino rats, males by gender, of Wistar strain, and of average weight 180 g, were used in our study. These animals were accommodated for one week in laboratory cages in our animal facility unit, provided with rat chow and water ad libitum, and subjected to natural light/dark cycles which equal 12 h/12 h. The experimental protocol was accepted by the Institutional Ethical Committee, Medicine Faculty, Minia University, Egypt, on 11/11/2024 (Approval No: 1348/11/2024), which is in line with the proper handling rules of laboratory animals prepared by the National Institute of Health and ARRIVE guidelines. Rats were separated randomly into 4 groups of 6 rats each. This sample size was determined according to the equation method “E value”, which is the degree of freedom of analysis of variance (ANOVA). E = Total number of animals − Total number of groups. If the E value lies between 10 and 20, the sample size is sufficient [36].

### 4.2. Induction of Type II Diabetes Mellitus

After fasting during the night, rats were injected once intraperitoneally (i.p) with nicotinamide (NA; dissolved in saline), firstly at a dose level of 100 mg/kg to reduce the destructive effect of streptozotocin (STZ; dissolved in 0.1 M citrate buffer with PH 4.5) on pancreatic β cells. STZ was injected once i.p at a dose level of 55 mg/kg 15 min after NA administration. After three days, the levels of fasting blood glucose for all rats were tested using glucose-oxidase reagent strips (Accu-Chek, Roche Inc., Indianapolis, IN, USA). Rats with blood glucose levels greater than 200 mg/dL were considered diabetic and incorporated in this study [37]. Experimental groups were distributed randomly into the following:

**1—Control (C) group**: Rats were injected once i.p with an equal volume of citrate buffer plus saline as vehicles for STZ and NA, respectively. These rats were left for 8 weeks without treatment.

**2—Type II diabetes mellitus (DM) group**: Diabetic rats were left for 8 weeks for induction of testicular injury, receiving equal volumes of DMSO (0.1%) as a vehicle for different treatments [37].

**3—DM + Ferrostatin-1 (DM + Fer-1)**: Diabetic rats were injected i.p with Fer-1 at a dose level of 10 mg/kg/day once every other day for 8 weeks [4].

**4—DM + Lipoxin A4 (DM + LXA4)**: Diabetic rats were injected i.p with LXA4 at a dose level of 10 µg/kg/day [12,30] for 8 weeks [38].

### 4.3. Treatment Protocol

All treatments were supplied by the Sigma Aldrich Company (St. Louis, MO, USA). LXA 4 and Fer-1 were dissolved in DMSO (0.1% diluted with saline) instantly before use [39,40]. The doses and the duration of different treatments were selected according to a preliminary study that is in line with previous studies; the selected dose of Fer-1 had the ability to inhibit ferroptosis and reduce spermatogenic dysfunction in the testicular tissue of diabetic mice [4], while the dose of LXA4 was chosen as it protected the testicular tissue against ischemia–reperfusion and also protected the erectile tissues of rats against the injurious effect of diabetes [12].

### 4.4. Samples Collection and Analysis

Twenty-four hours from the last injection, after eight hours of fasting, rats were euthanized using sodium phenobarbital anesthesia at a dose level of 25 mg/kg to collect blood samples and to separate sera that were stored at −20 °C till the time of analysis. Colorimetric kits were used for assays of serum levels of glucose, total cholesterol, triglycerides (Biodiagnostics, Giza, Egypt) and total iron (MyBiosource, Inc., San Diego, CA, USA), while ELISA kits were used for assays of insulin and testosterone (MyBiosource, Inc., San Diego, CA, USA).

The scrotum of every single rat was opened. The testes were removed and weighed. The right testis was stored at −80 °C for RT-PCR analysis and biochemical analysis. Testicular tissue homogenates were prepared according to kit instructions for the colorimetric analysis of lipid peroxides (MDA), glutathione (Biodiagnostics, Giza, Egypt), and total iron (MyBiosource, Inc., San Diego, CA, USA), while ELISA kits were used for assays of reactive oxygen species (ROS) concentration, *NF-κB p65*, ferritin, glutathione peroxidase 4 (*GPX4*), tumor necrosis factor alpha (*TNF-α*), and interleukin- 37 (*IL-37*) (MyBiosource, Inc., San Diego, CA, USA). The left testis was immersed in formalin for histological and immunohistochemical studies.

### 4.5. Assessment of Sperm Count and Motility

The cauda epididymis of every single rat was cautiously separated, cut into pieces, and minced instantly in physiological saline (5 mL), then incubated at 37 °C for 30 min to let sperms leave the epididymis. The percentage of motile sperms was estimated by an ordinary microscope at ×400 magnification. The sperm count per ml was estimated using a hemocytometer [41].

### 4.6. Real Time PCR Assessment of GPX4

Testicular tissue homogenates were cryopreserved at −80 °C prior to analysis. The Direct-zol RNA Miniprep Plus Kit (Cat# R2072; Zymo Research, Irvine, CA, USA) was used to isolate total RNA from TRIzol^®^-lysed samples, following the manufacturer’s protocol. This method facilitates comprehensive RNA recovery, including small RNA species, through ethanol-mediated binding to Zymo-Spin™ columns. Residual genomic DNA was enzymatically digested via on-column DNase I treatment. Post-wash cycles eluted the purified RNA in nuclease-free water. RNA integrity and concentration were quantified spectrophotometrically (Beckman DU Series, Brea, CA, USA) at 260 nm, with 260/280 nm ratios confirming purity.

Complementary DNA (cDNA) synthesis and amplification were conducted in a unified reaction using the SuperScript IV One-Step RT-PCR Kit (Cat# 12594100; Fisher Scientific, Waltham, MA, USA). Quantitative PCR was executed with GoTaq PCR Master Mix (Promega, Madison, WI, USA) on an Applied Biosystems Phase One Real-Time PCR System (Foster City, CA, USA). Thermal cycling parameters comprised an initial denaturation (95 °C, 2 min), followed by 40 cycles of denaturation (95 °C, 15 s), combined annealing/extension (60 °C, 1 min), and a terminal extension (60 °C, 30 s). Amplification specificity was confirmed via melt curve analysis.

Gene expression quantification of GPX4 was standardized against the endogenous control glyceraldehyde-3-phosphate dehydrogenase (*GAPDH*). The relative mRNA levels were estimated using the comparative threshold cycle (2^−ΔΔCT^) method, with triplicate reactions ensuring analytical reproducibility. This methodology ensures precise quantification of redox regulatory gene expression while maintaining RNA integrity and minimizing genomic DNA contamination. The primer sequences used are as follows (Table 4):

### 4.7. Western Blotting Analysis of Nrf2 and SLC7A11

Testicular tissues were homogenized, and total protein was extracted using the Ready Prep™ Extraction Protein Kit (Bio-Rad, Cat. #163-2086, Hercules, CA, USA) in conformity with the manufacturer’s guidance. The concentrations of proteins were determined using the Bradford method with a kit from Bio Basic Inc. (SK3041, Markham, ON, Canada), according to the established protocol by [42]. Each protein sample (20 µg) was mingled with an equal volume of Laemmli buffer and denatured by heating at 95 °C for 5 min.

Proteins (7.5 µg per sample) were dissociated via SDS-PAGE and then transferred onto PVDF membranes. The membrane was blocked for 1 h at room temperature using 3% BSA in TBST to prevent non-specific binding [43]. After blocking, the membrane was incubated for 12 h at 4 °C with primary antibodies diluted in TBST, including mouse monoclonal *β-actin* (1:2000; Elabscience, Houston, TX, USA, #E-AB-20031), rabbit monoclonal *Nrf2* (1:1000; Cell Signaling Technology, Danvers, MA, USA, #20733), and anti-*SLC7A11* (AA 215–264) (Antibodies Online, Limerick, PA, USA, #ABIN6743218).

After primary antibody incubation, membranes were washed three to five times (5 min each) with TBST and then incubated for one hour at room temperature with horseradish peroxidase (HRP)-conjugated secondary antibodies (Goat anti-rabbit IgG-HRP; Novus Biologicals, Centennial, CO, USA).

After additional TBST washes, the bands of protein were visualized using the Clarity™ Western ECL Substrate (Bio-Rad, Hercules, CA, USA), and signals were verified using a CCD camera-based imaging system.

Band intensities were assessed using the ChemiDoc MP Imaging System (Bio-Rad, Hercules, CA, USA) and associated software. Expression levels of target proteins were normalized against β-actin, and data were analyzed relative to the control group.

### 4.8. Histological and Light Microscopic Study

Testis specimens were fixed in a solution of buffered formalin (10%), and tissues were dealt with to get the paraffin blocks. Following the preparation of paraffin blocks, sections (5–7 μm) were stained with the stains of hematoxylin and eosin (H&E) [44] and Perl’s Prussian blue (for the evaluation of the Fe^3+^ (ferric iron) content) [45].

Immunohistochemical staining was carried out as stated in the manufacturer’s instructions. To sum up, sections were deparaffinized, rehydrated, and pretreated with hydrogen peroxide (0.01%) to stop the activity of endogenous peroxidase. Then, sections were submerged in citrate buffer (0.01 M, PH. 6) for a time of 10 min for unmasking the antigenic site. After that, the antigen was restored in the EDTA buffer using a microwave for a period of 20 min. Later, sections were incubated in primary antibodies through the night at 4 °C. The primary antibodies used were anti-high-mobility group box 1 protein (HMGB1) antibody (proinflammatory marker, ab208282, 1:200; Abcam, Waltham, MA, USA) and anti-Tunnel antibody (apoptotic marker, ab11420, 1:500; Abcam). Afterwards, the sections were incubated for a period of 1 h in avidin–biotin complex and washed and incubated in peroxidase substrate (DAB) solution for another period of 10 min. Eventually, counterstaining of sections with hematoxylin was carried out. This was followed by dehydrating sections in absolute alcohol and clearing them with xylene and, finally, they were mounted. Immunoreactivity was visualized as dark brown cytoplasmic and nuclear staining. HMGB1 expression is nuclear and cytoplasmic, but Tunnel expression is cytoplasmic. Positive and negative control slides were prepared. The negative control slides were prepared by the same previous methods without adding the primary antibodies [46].

H&E-, Perl’s Prussian blue-, and immunohistochemically stained slides were photographed in the Histology and Cell Biology Department, Medicine Faculty of Minia University, using a high-resolution color-digital camera fixed on a BX51-microscope (Olympus, Tokyo, Japan), connected to a computer operating the LC-micro-application software V2.2.

### 4.9. Data and Statistical Analysis

To diminish bias and assert the validity of our results, the researchers were blind to the distribution of the different groups of our study, starting from treatment administration to the collection of data and its analysis. Data was analyzed by Graph Pad Prism (version 8, Graph Pad Software, San Diego, CA, USA). For the parameters of each group, the mean and standard error of the mean (SEM) were determined. One-way ANOVA followed by Tukey’s post hoc test was performed for multiple comparisons between groups to detect any significant differences. Statistics are significant for *p*-values equal to or below 0.05.

## 5. Conclusions

LXA4 mitigated testicular ferroptosis in diabetic rats and improved the endocrine and spermatogenic functions of the testis via enhancing the antioxidant *Nrf2/SLC7A11/GPX4* pathway. However, to the best of our knowledge, this is the first study that demonstrates a link between LXA4 and the regulation of ferroptosis in the testicular tissue of diabetic rats, and thus, the results reported herein have to be seen in the light of some limitations that could be targeted in future research. Here, we studied the role of LXA4 in vivo using only a one-dose regimen of LXA4 without dose–response assessment, which may limit the strength of direct comparison with Fer-1. Thus, further studies are highly recommended to examine the effect of different doses of LXA4 in such a model with different administration regimens to optimize the benefits, and clinical research on diabetic patients should also be considered.

## Figures and Tables

**Table 1:** Evaluation of serum metabolic parameters.

Parameters		Groups		
	C	DM	DM + Fer-1	DM + LXA4
Glucose (mg/dL)	82.67 ± 2.6 ^a^	275.5 ± 10.4 ^b^	123.2 ± 2 ^c^	149.8 ± 5.6 ^d^
Insulin (µIU/mL)	7.83 ± 0.26 ^a^	3.88 ± 0.23 ^b^	6.75 ± 0.29 ^c^	5.53 ± 0.15 ^d^
Total cholesterol (mg/dL)	108.8 ± 1.14 ^a^	229.7 ± 4.8 ^b^	123.3 ± 2.98 ^c^	144 ± 2.9 ^d^
Triglycerides (mg/dL)	87.7 ± 3.8 ^a^	157 ± 3.78 ^b^	109.8 ± 2.9 ^c^	127.7 ± 2.56 ^d^

Data shown are for mean ± S.E. of six rats. Means in the same horizontal row with different letters (a, b, c and d) indicate significant differences between groups (*p* value ≤ 0.05). Pairwise comparisons using Tukey’s post hoc test revealed significant differences between all groups. All comparisons (*p* < 0.0001) except for glucose; a versus c (*p* = 0.0008) and c versus d (*p* = 0.029), insulin; a versus c (*p* = 0.02), b versus d (*p* = 0.0005) and c versus d (*p* = 0.009), total cholesterol; a versus c (*p* = 0.02), and c versus d (*p* = 0.001) and triglycerides; a versus c (*p* = 0.0007) and c versus d (*p* = 0.0055). C: control; DM: type II diabetes group; Fer-1: ferrostatin-1; LXA4: lipoxin A4.

**Table 2:** Evaluation of serum iron and testicular tissue levels of iron and ferritin.

Parameters		Groups		
	C	DM	DM + Fer-1	DM + LXA4
Serum iron (nmol/µL)	0.012 ± 0.001 ^a^	0.10 ± 0.01 ^b^	0.038 ± 0.002 ^c^	0.062 ± 0.001 ^d^
Tissue iron (nmol/mg tissue)	0.15 ± 0.02 ^a^	0.38 ± 0.01 ^b^	0.22 ± 0.018 ^c^	0.29 ± 0.005 ^d^
Tissue ferritin (ng/mg tissue)	35.8 ± 1.45 ^a^	8 ± 0.86 ^b^	25.8 ± 1.25 ^c^	18.2 ± 0.95 ^d^

Data shown are for mean ± S.E. of six rats. Means in the same horizontal row with different letters (a, b, c and d) indicate significant differences between groups (*p* value ≤ 0.05). Pairwise comparisons using Tukey’s post hoc test revealed significant difference between all groups. All comparisons (*p* < 0.0001) except for serum iron; a versus c (*p* = 0.02), b versus d (*p* = 0.0006) and c versus d (*p* = 0.04), tissue iron; a versus c (*p* = 0.004), b versus d (*p* = 0.0004) and c versus d (*p* = 0.005), and tissue ferritin; c versus d (*p* = 0.0007). C: control; DM: type II diabetes group; Fer-1: ferrostatin-1; LXA4: lipoxin A4.

**Table 3:** Evaluation of testicular oxidative and inflammatory status.

Parameters		Groups		
	C	DM	DM + Fer-1	DM + LXA4
ROS (U/mg tissue)	102.5 ± 3.8 ^a^	396.5 ± 3.5 ^b^	139.3 ± 2.72 ^c^	174 ± 5 ^d^
MDA (nmol/mg tissue)	34.34 ± 1. 5 ^a^	97.13 ± 3.6 ^b^	47.36 ± 2 ^c^	61.9 ± 1.9 ^d^
GSH (mg/mg tissue)	125.7 ± 1.63 ^a^	55.93 ± 1.56 ^b^	112.6 ± 2.49 ^c^	91.5 ± 2.26 ^d^
GPX4 (ng/mg tissue)	48.82 ± 1.45 ^a^	21.83 ± 1.35 ^b^	42 ± 1.37 ^c^	36.33 ± 0.88 ^d^
TNF-α (pg/mg tissue)	89.67 ± 1.36 ^a^	170.7 ± 1.91 ^b^	113.8 ± 3.46 ^c^	129.7 ± 1.65 ^d^
IL-37 (pg/mg tissue)	314.2 ± 4 ^a^	118 ± 4.2 ^b^	283.7 ± 3.8 ^c^	260.2 ± 4.6 ^d^
NF-κB p65 (pg/mg tissue)	120.3 ± 2.71 ^a^	229.5 ± 3.42 ^b^	146.8 ± 2.96 ^c^	161.3 ± 2.33 ^d^

Data shown are for mean ± S.E. of six rats. Means in the same horizontal row with different letters (a, b, c and d) indicate significant differences between groups (*p* value ≤ 0.05). Pairwise comparisons using Tukey’s post hoc test revealed significant differences between all groups. All comparisons (*p* < 0.0001) except for MDA; a versus c (*p* = 0.005) and c versus d (*p* = 0.025), GSH; a versus c (*p* = 0.001), GPX4; a versus c (*p* = 0.006) and c versus d (*p* < 0.001), TNF-α; c versus d (*p* = 0.0004), IL-37; a versus c (*p* = 0.0003) and c versus d (*p* = 0.003) and NF-κB p65; c versus s d (*p* = 0.009). C: control; DM: type II diabetes group; Fer-1: ferrostatin-1; LXA4: lipoxin A4; ROS: reactive oxygen species; MDA: malondialdehyde; GSH: glutathione; GPX4: glutathione peroxidase 4; TNF-α: tumor necrosis factor alpha; IL-37: interleukin-37; NF-κB p65: nuclear factor NF-kappa-B p65 subunit.

**Table 4:** Oligonucleotide primer sequences used for qPCR.

	Forward Sequence	Reverse Sequence
*GPX-4*	5′GGCTACAATGTCAGGT3′	5′TTATCAATGAGAAACTTGGTAA3′
*GAPDH*	5′TGGATTTGGACGCATTGGTC3′	5′TTTGCACTGGTACGTGTTGAT3′

## Data Availability

The original contributions presented in this study are included in the article. Further inquiries can be directed to the corresponding authors.
